# Integrative analysis confirms TPX2 as a novel biomarker for clinical implication, tumor microenvironment, and immunotherapy response across human solid tumors

**DOI:** 10.18632/aging.205498

**Published:** 2024-02-02

**Authors:** Mingxia Zhu, Xiaping Wang, Qing Zhang, Chen Xie, Tongshan Wang, Kai Shen, Lan Zhang, Xin Zhou

**Affiliations:** 1 Department of Radiation Oncology, The First Affiliated Hospital of Soochow University, Suzhou 215006, China; 2 Department of Pathology, The Second Affiliated Hospital of Nanjing Medical University, Nanjing 210000, China; 3 Department of Neurosurgery, Xinghua People’s Hospital, Xinghua 225700, China; 4 Department of Gastroenterology, The First Affiliated Hospital of Soochow University, Suzhou 215006, China; 5 Department of Oncology, First Affiliated Hospital of Nanjing Medical University, Nanjing 210029, China; 6 Department of Radiation Oncology, Shanghai Tenth People’s Hospital of Tongji University, Shanghai 200072, China

**Keywords:** TPX2, pan-cancer, single-cell RNA-Seq, prognosis, immunotherapy

## Abstract

Targeting Protein for Xenopus Kinesin Like Protein 2 (TPX2) serves as a microtubule associated protein for the regulation of spindle assembly and tumorigenesis. We aim to investigate the prognostic and immunological role of TPX2 in pan-cancer. TCGA database, Tumor Immune Single-cell Hub (TISCH), and Human Protein Atlas (HPA) were retrieved to evaluate the expression pattern of TPX2 as well as its diagnostic and prognostic value in solid tumors. Genomic alterations of TPX2 were assessed with cBioPortal database. *In vitro* experiments in lung adenocarcinoma (LUAD) were performed to confirm the potential role of TPX2. Overexpression of TPX2 was found in 22 types of cancers, and was positively related with copy number variations (CNV) and negative with methylation. Up-regulated TPX2 could predict worse outcomes in the majority of cancers. Single-cell analysis revealed that TPX2 was mainly distributed in malignant cells (especially in glioma) and proliferating T cells. Genomic alteration of TPX2 was common in different types of tumors, while with prognostic value in two types of cancers. Additionally, significant correlations were found between TPX2 expression and tumor microenvironment (including stromal cells and immune cells) as well as immune related genes across cancer types. Drug sensitivity analysis revealed that TPX2 could predict response to chemotherapy and immunotherapy. Functional analyses demonstrated close relationship of TPX2 with immune function and malignant phenotypes. Finally, it was confirmed that knockdown of TPX2 could reduce proliferation and migration ability of LUAD cells. In summary, TPX2 could serve as a diagnostic and prognostic biomarker and a potential immunotherapy marker.

## INTRODUCTION

Targeting Protein for Xenopus Kinesin Like Protein 2 (TPX2) acts as a microtubule-associated protein involved in mitotic spindle assembly, primarily by activating the cell cycle kinase protein Aurora A [1]. Overexpression of TPX2 can cause centrosome amplification, leading to DNA polyploidy and facilitating tumorigenesis [2, 3]. Extensive studies have revealed that TPX2 is upregulated and related to poor prognosis in multiple solid tumors, such as breast cancer and hepatocellular carcinoma [4–7]. However, to our knowledge, many studies on TPX2 mainly focused on a specific cancer type. The study conducted by Shao et al. only provided limited information on TPX2 in pan-cancer [8]. Consequently, more systematic analysis based on large clinical data is required to identify the role of TPX2.

The tumor microenvironment is instrumental in the initiation and progression of malignancies. Throughout their lifetime, tumor cells are monitored by immune cells. The lack of immune cells capable of eradicating preneoplastic cells can precipitate tumor development and progression [9, 10]. Furthermore, emerging evidence increasingly supports that infiltrating immune cells can significantly impact tumor outcomes and prognoses [11, 12]. Recently, immune checkpoint inhibitors (ICIs) have demonstrated remarkable efficacy in treating multiple types of advanced cancer. Unfortunately, this treatment is beneficial to only a limited subset of patients [13, 14]. Thus, it is crucial to explore tumor-immune interaction and identify reliable biomarkers for immunotherapy.

A comprehensive investigation of TPX2 expression profiles, diagnostic and prognostic landscape was conducted using the TCGA database. Further, we examined the relationship of TPX2 expression with clinical features, and the tumor microenvironment. Other parameter analyses, such as gene alteration, DNA methylation, single-cell analysis, drug sensitivity, and the ceRNA network analysis of TPX2 were also explored in our study. Lastly, we verified TPX2 expression at both mRNA and protein levels in lung cancer tissues as well as explored biological functions in a lung cancer cell line.

## RESULTS

### TPX2 gene expression and diagnostic value in pan-cancer

**Figure 1:**
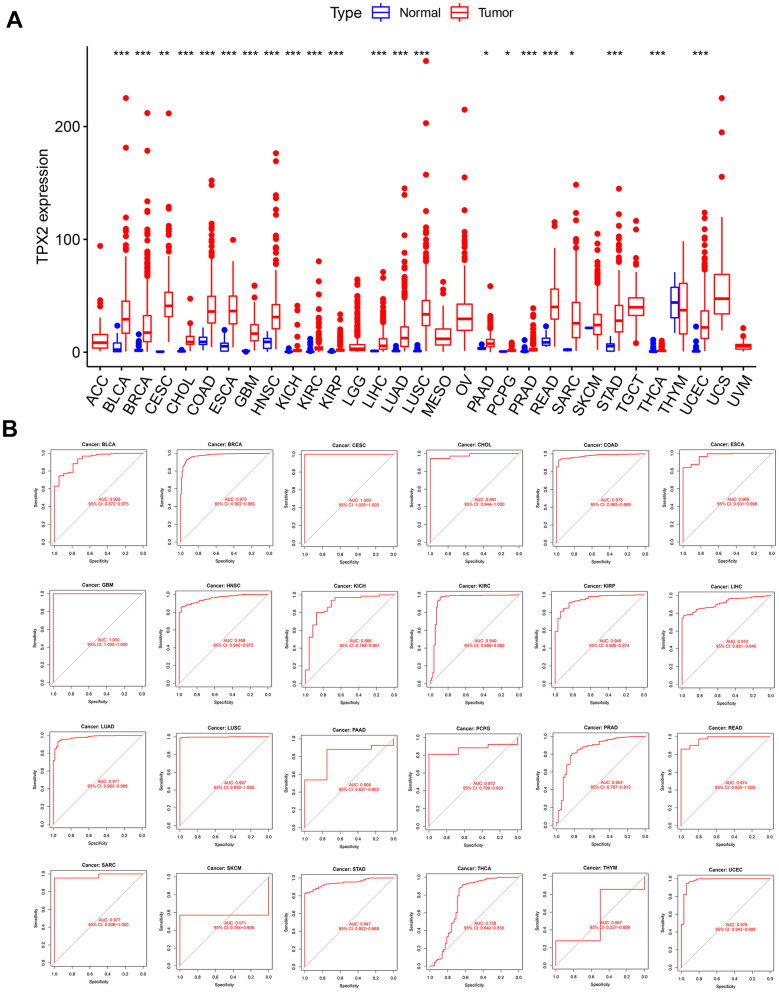
**Expression level and diagnostic value of TPX2 in pan-cancer from the TCGA database.** (**A**) Boxplot of the mRNA expression of TPX2 in 31 solid cancer types from the TCGA database. Expression level of TPX2 was presented as FPKM. (**B**) ROC curve analyses to determine the diagnostic value of TPX2 in the TCGA database. *p< 0.05, **p< 0.01, ***p< 0.001.

Initially, 31 types of solid tumors were analyzed for TPX2 mRNA expression. There were 22 tumors with higher expression of TPX2 than in the corresponding non-carcinoma tissues (Figure 1A). Next, we evaluated the diagnostic value of TPX2 using ROC curve. TPX2 can be used as a good diagnostic marker for the above 22 tumor types (Figure 1B).

### Single-cell expression analysis of TPX2

**Figure 2:**
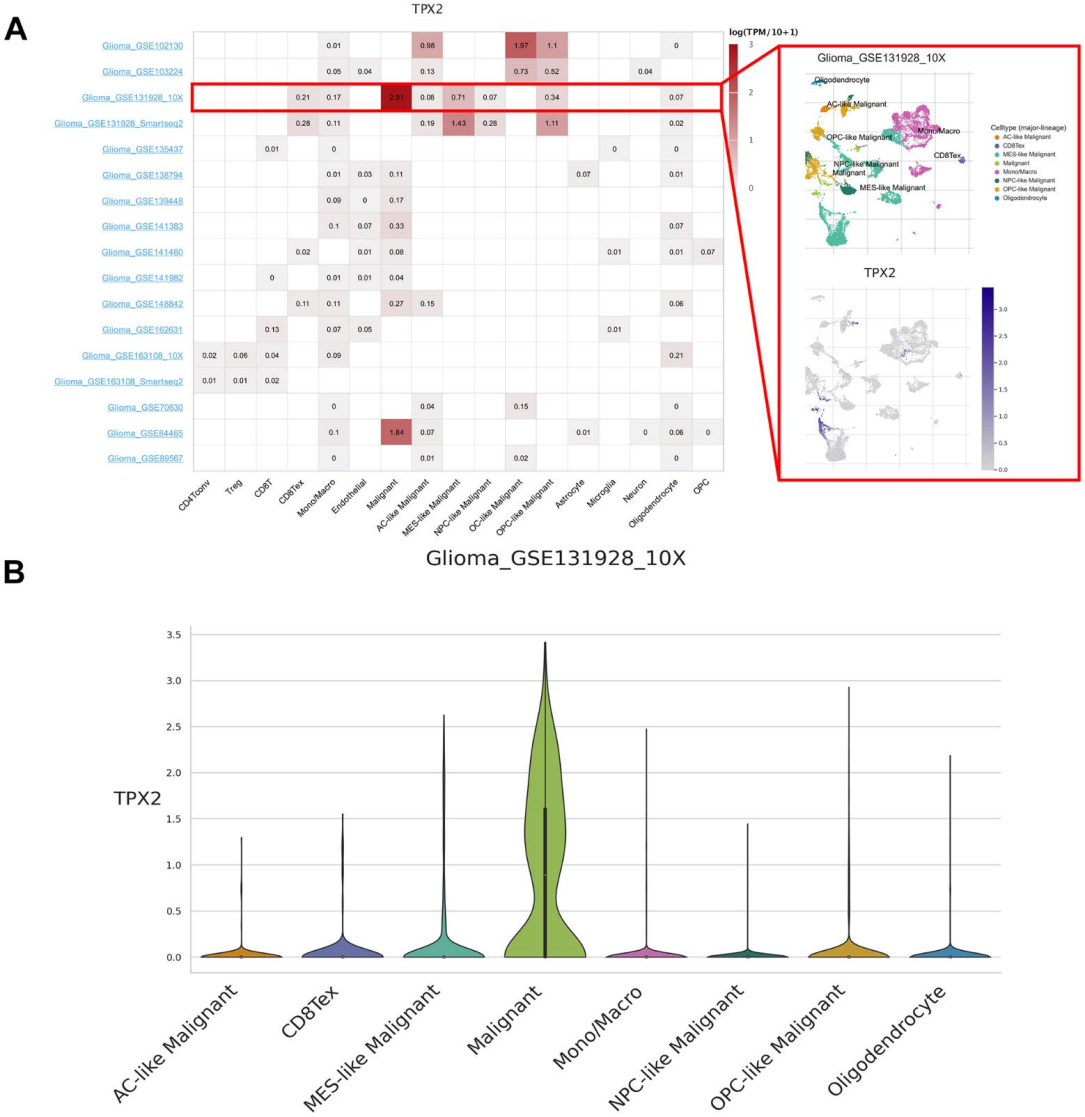
**Single-cell expression analysis of TPX2 in glioma.** (**A**) Heatmap of TPX2 expression in 17 glioma datasets (red rectangle: GSE131928_10× glioma dataset). (**B**) The violin plot showed the overexpression of TPX2 in malignant cells in GSE131928_10× glioma dataset.

We analyzed 190 single-cell datasets of cancer samples in order to identify the main cell types expressing TPX2 in the cancer microenvironments. TPX2 expression was up-regulated in malignant cells of the glioma microenvironment. In particular, in the glioma GSE131928 10× dataset, TPX2 was primarily expressed in malignant cells compared with other cells (Figure 2). High levels of TPX2 expression were observed in proliferating T cells in the microenvironment of KIRC, BRCA, OV, NSCLC, CRC, ESCA, LIHC, SKCM and THCA, while it was lost in malignant cells (Supplementary Figure 1).

### DNA methylation and protein phosphorylation analysis of TPX2

**Figure 3:**
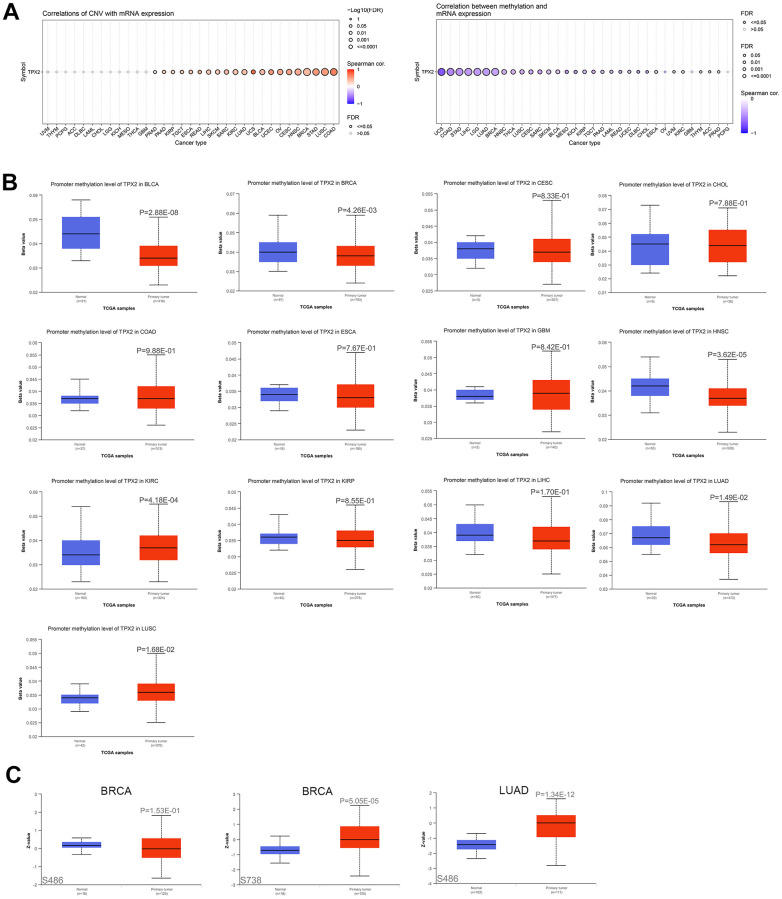
**The association of TPX2 expression and CNV, DNA methylation, protein phosphorylation.** (**A**) The association of CNV and expression of TPX2 mRNA using the GSCA analysis (Left). The association of DNA methylation and expression of TPX2 mRNA using the GSCA analysis (Right). Red dots indicate positive association. Blue dots indicate negative association. (**B**) The promoter methylation level of TPX2 between primary tumor and normal tissues in different cancer types using the GSCA analysis. (**C**) The phosphorylation level of TPX2 protein between primary tumor and normal tissues in BRCA and LUAD using CPTAC analysis. p-value < 0.05 was considered statistically significant.

Based on Gene Set Cancer Analysis (GSCA), we found that TPX2 copy number variation (CNV) was positively related to its mRNA expression in different cancers, suggesting that CNV could be a factor in the modification of TPX2. Negative correlations between TPX2 methylation and TPX2 expression were observed across multiple human cancers (Figure 3A). Promoter hypermethylation of TPX2 occurred in KIRC and LUSC, while hypomethylation occurred in BLCA, BRCA, HNSC, and LUAD (Figure 3B). Using the Clinical Proteomic Tumor Analysis Consortium (CPTAC) analysis, significantly elevated levels of TPX2 phosphorylation at S738 were identified in primary BRCA compared to control tissues. Phosphorylation levels at S486 were significantly elevated in primary LUAD. A low level of phosphorylation at S486 was also observed in BRCA, but not statistically significant (Figure 3C). However, the molecular mechanism of TPX2 phosphorylation levels in tumors needs to be further investigated.

### TPX2 protein expression and its localization

**Figure 4:**
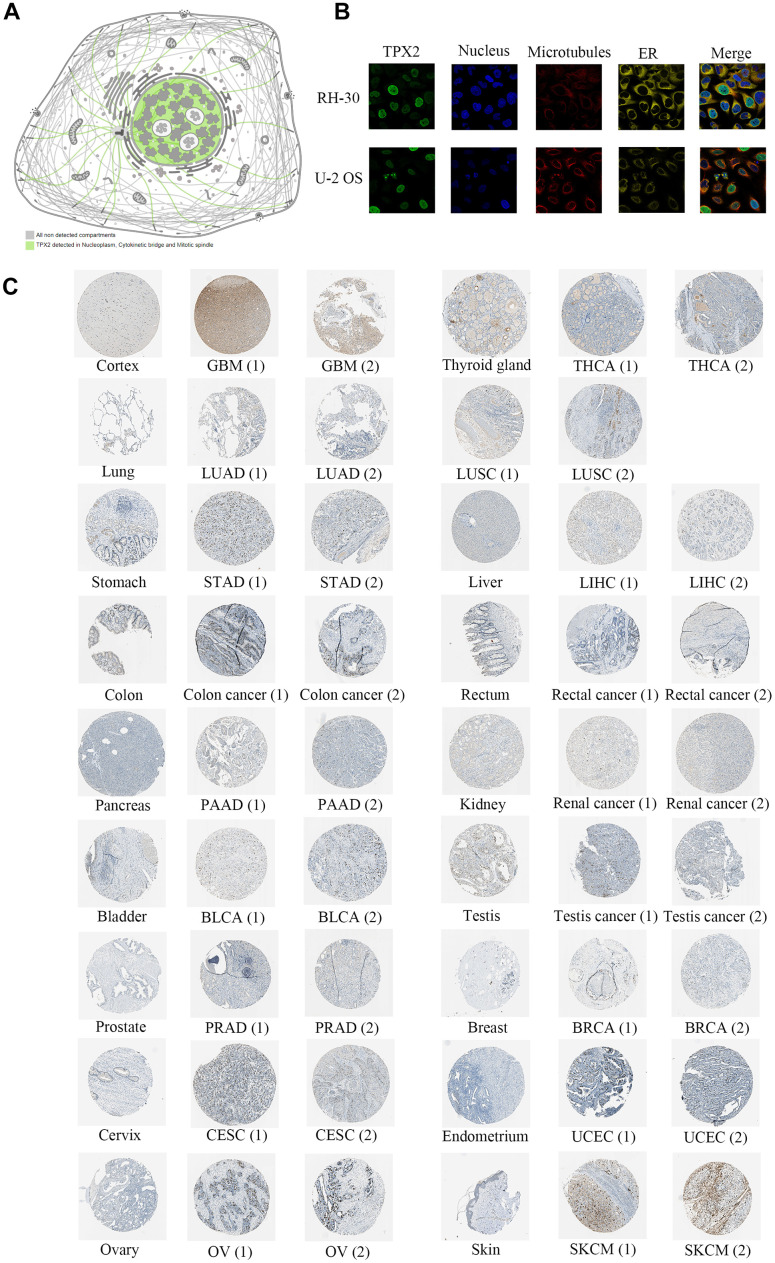
**Protein expression analysis of TPX2 in the HPA database.** (**A**) TPX2 was detected in nucleoplasm, cytokinetic bridge and mitotic spindle. (**B**) Immunofluorescence staining of the subcellular localization of TPX2 in RH-30 and U-2 OS tumor cells. (**C**) Representative immunohistochemical staining of TPX2 in normal and tumor tissues of different cancer types.

The Human Protein Atlas (HPA) cohort was used to investigate TPX2 protein expression. As shown in Figure 4A, TPX2 was detected in nucleoplasm, cytokinetic bridge and mitotic spindle. Consistently, according to immunofluorescence images, the TPX2 protein was mostly distributed in nucleoplasm of RH-30 and U-2 OS tumor cells (Figure 4B). A total of 17 tumor tissues (https://www.proteinatlas.org/ENSG00000088325-TPX2/pathology) expressed higher levels of TPX2 protein than normal tissues (https://www.proteinatlas.org/ENSG00000088325-TPX2/tissue) (Figure 4C).

### Genetic alteration analysis of TPX2

**Figure 5:**
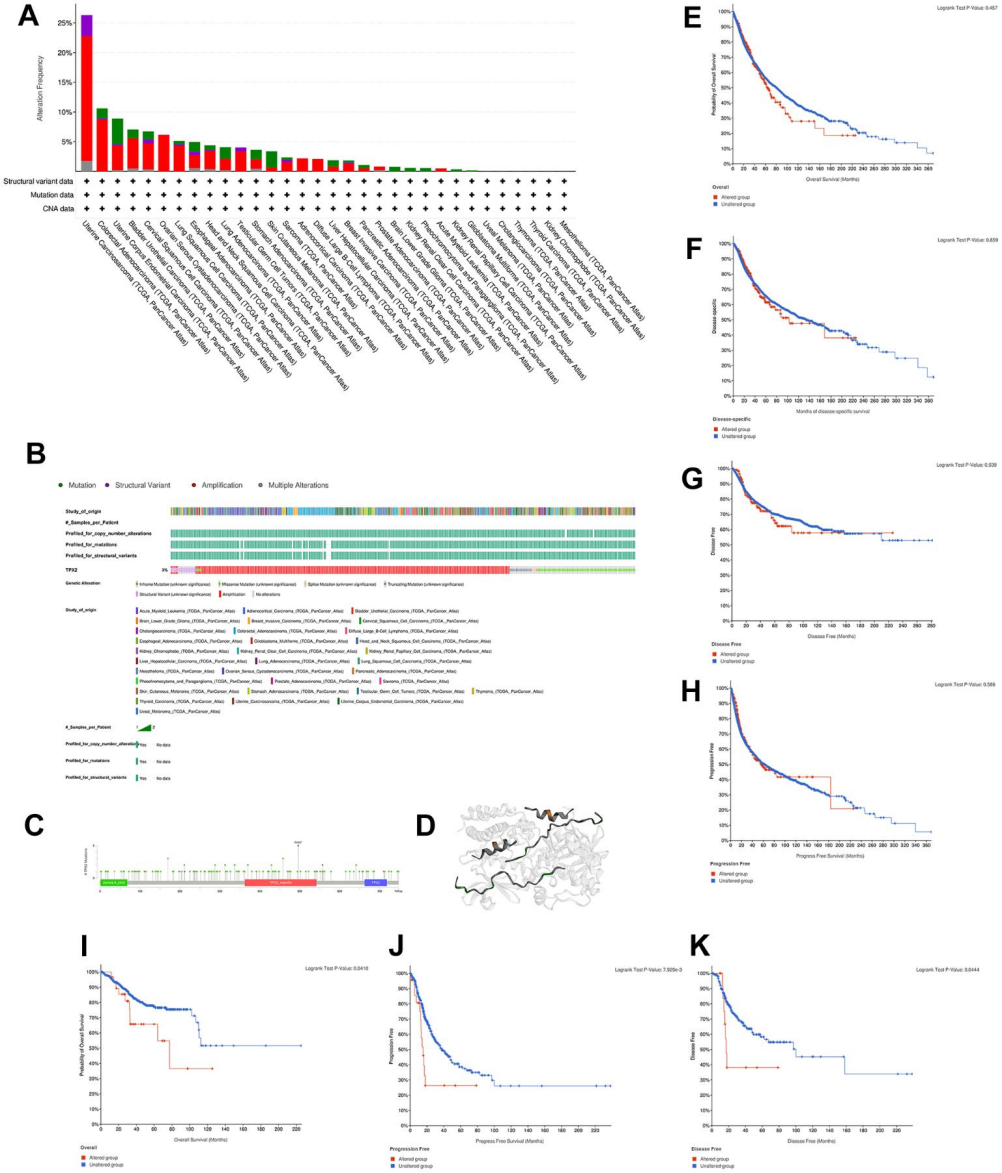
**Mutation characteristic of TPX2 in different cancer types.** (**A**) The frequency of TPX2 mutations with mutation type across TCGA cancers by cBioPortal. Red represents amplicaion. Green represents mutation. Purple represents structural variant. Grey represents multiple alterations. (**B**) OncoPrint visual summary of alterations in a query of TPX2 by cBioPortal. (**C**) Mutation site of TPX2 displayed by cBioPortal. (**D**) Corresponding 3D structures of TPX2 displayed by cBioPortal. (**E**–**H**) The associations of pan-cancer TPX2 mutation status with OS, DSS, DFS and PFS by cBioPortal. (**I**–**K**) The associations of TPX2 mutation status with OS of UCEC patients (**I**) and survival of LUSC patients ((**J**) PFS; (**K**) DFS) by cBioPortal. p-value < 0.05 was considered statistically significant.

We sought to explore the genetic alteration of TPX2 in TCGA tumor samples with cBioportal. Most cancer types exhibit TPX2 amplification patterns, particularly in UCS, which had more than 20% mutations with TPX2 amplification (Figure 5A). Oncoprint in cBioPortal showed the proportion and distribution of samples that have altered TPX2 factors (Figure 5B). We showed the mutation site of R496 in importin domain, which has the highest change frequency (Figure 5C). The 3D structure of the TPX2 protein was shown in Figure 5D. Then, we aimed to investigate whether the alteration TPX2 affects prognosis in different cancers. We observed no significant correlation between TPX2 mutation and OS, DSS, DFS, PFS when compared in pan-cancer (Figure 5E–5H). However, patients with alteration of TPX2 had poor OS compared to the unaltered group in UCEC, and poor PFS and DFS in LUSC (Figure 5I–5K).

### Prognostic value of TPX2 in various cancers

**Figure 6:**
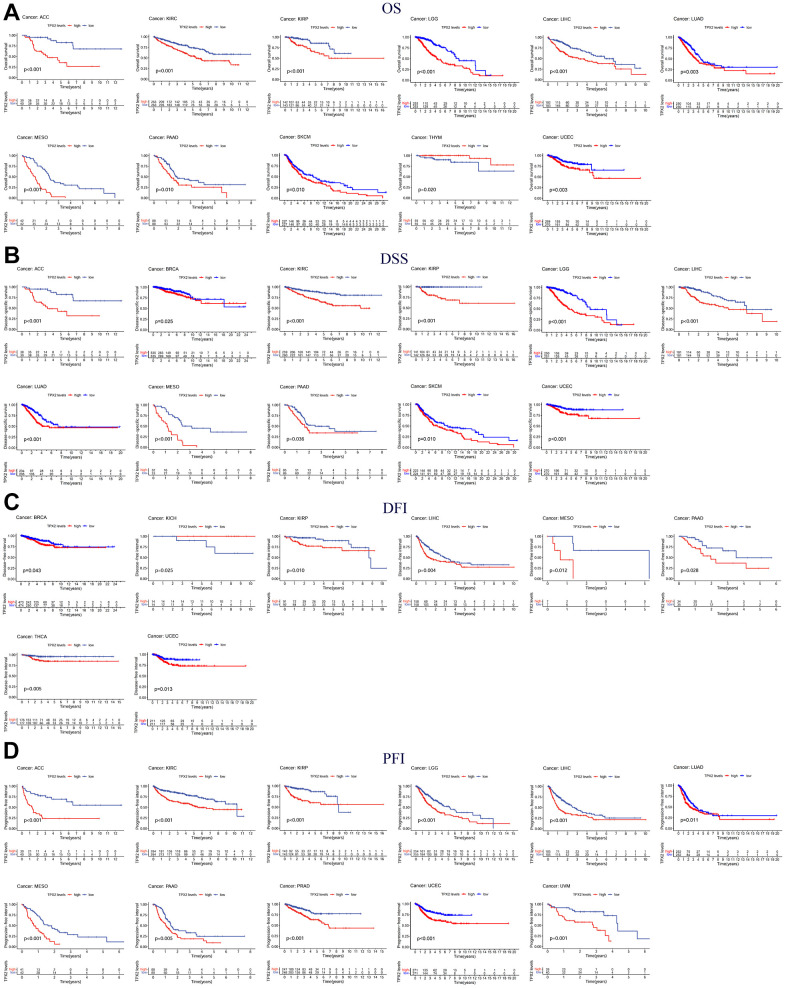
**Kaplan-Meier survival curves comparing the expression of TPX2 in pan-cancer from the TCGA database.** (**A**) Kaplan–Meier OS curves of TPX2 in 11 cancer types; (**B**) Kaplan–Meier DSS curves of TPX2 in 11 cancer types; (**C**) Kaplan–Meier DFI curves of TPX2 in 8 cancer types; (**D**) Kaplan–Meier PFI curves of TPX2 in 11 cancer types. p-value < 0.05 was considered statistically significant.

The association of TPX2 expression and prognosis was further investigated. Higher TPX2 expression was associated with worse OS in 10 tumor types, while in THYM, TPX2 up-regulation was related to improved OS. Like the OS analysis, high expression of TPX2 was statistically associated with poor DSS. For DFI, high TPX2 expression was related to poor DFI in 9 tumor types. Eventually, with regard to PFI, in 11 tumor types, high TPX2 levels were associated with poor prognosis (Figure 6).

**Figure 7:**
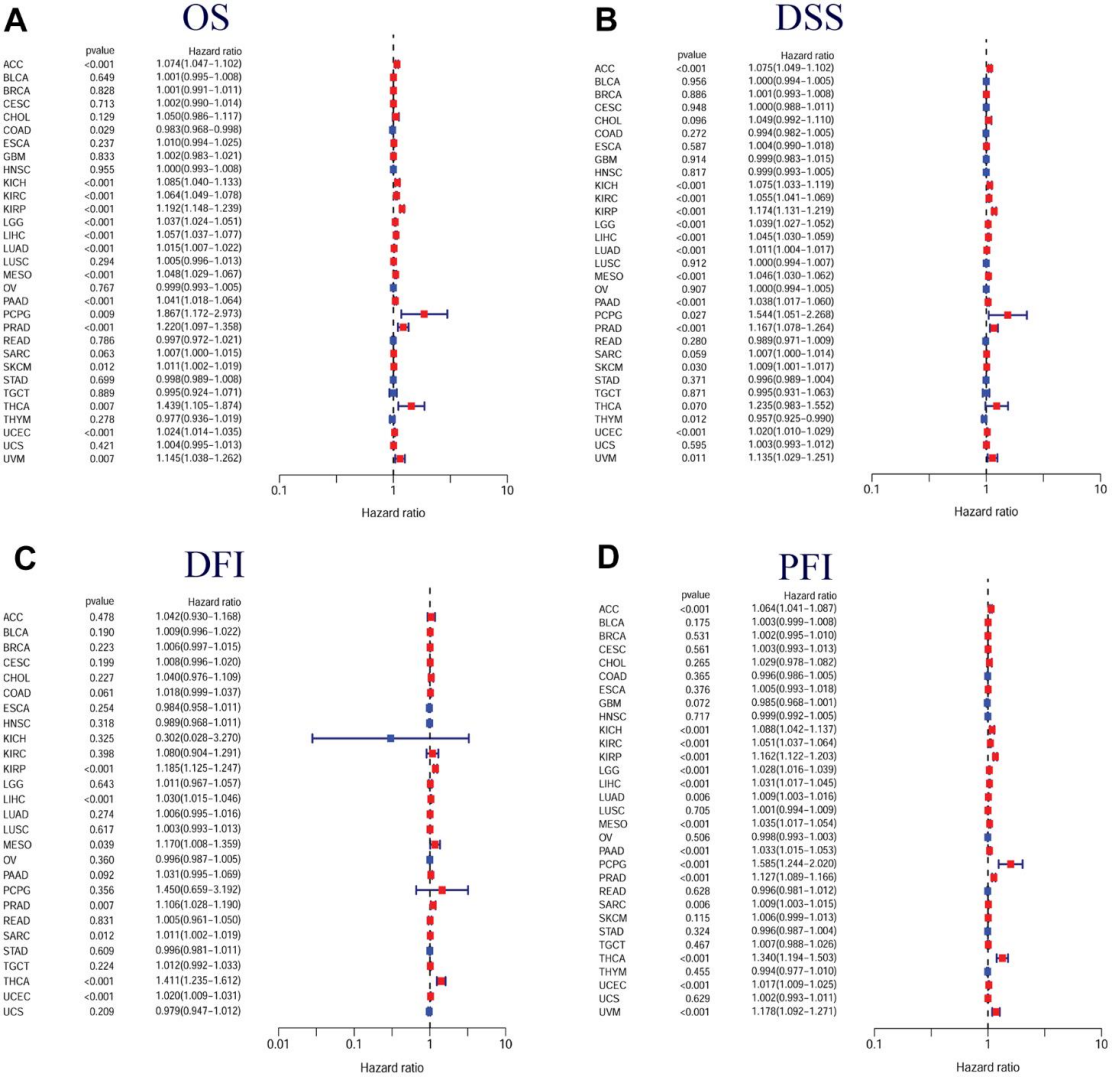
**Forest plots of in pan-cancer from the TCGA database.** (**A**) Relationship between TPX2 expression and OS; (**B**) Relationship between TPX2 expression and DSS; (**C**) Relationship between TPX2 expression and DFI; (**D**) Relationship between TPX2 expression and PFI. p-value < 0.05 was considered statistically significant.

Furthermore, Cox regression demonstrated that TPX2 expression could significantly impact the OS of 16 tumor types. In 15 tumor types, high TPX2 expression was a risk factor, while in COAD, it was protective. For DSS, similar results were found. Cox regression analysis in DFI demonstrated that in 7 tumor types, TPX2 was a high-risk factor. In addition, we also evaluated PFI and confirmed that in 15 tumor types, TPX2 was a high-risk gene (Figure 7).

### The association of TPX2 expression with clinical parameters

**Figure 8:**
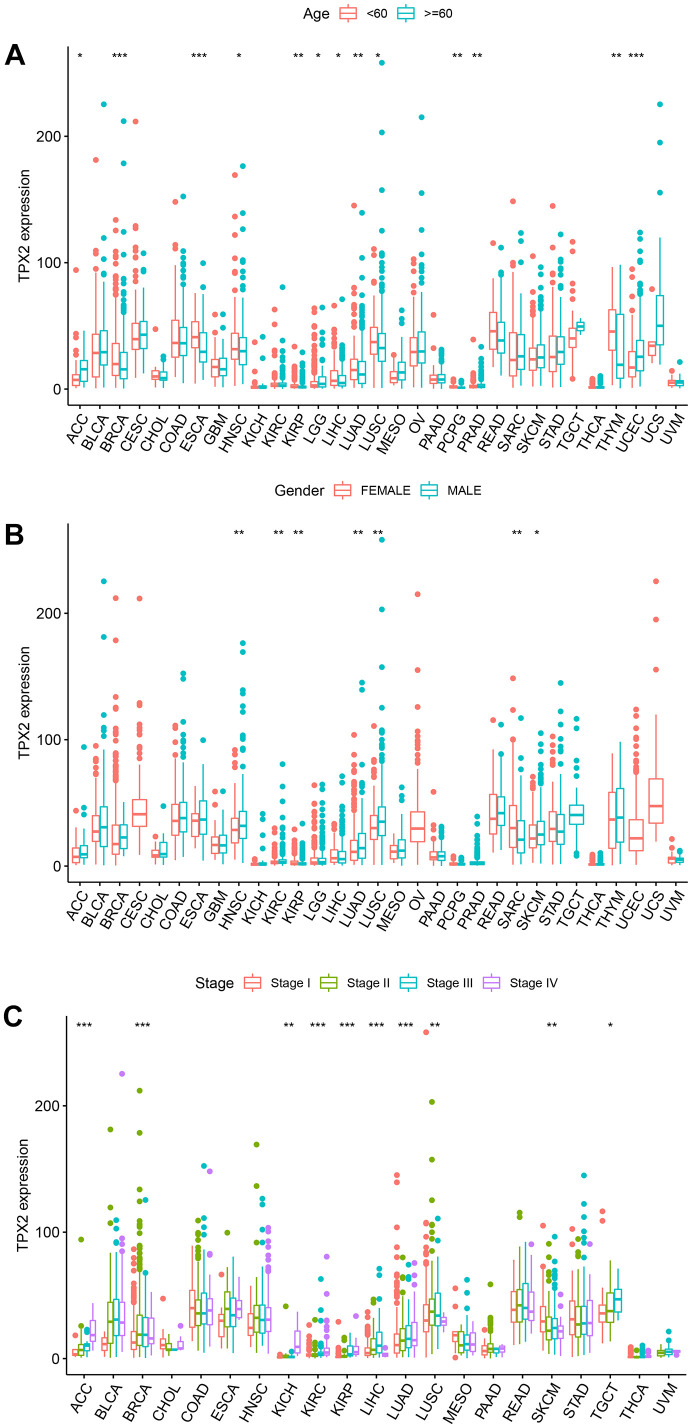
**The relationship between TPX2 expression and clinical parameters in various cancers from TCGA database.** (**A**) The relationship between TPX2 expression and age. (**B**) The relationship between TPX2 expression and gender. (**C**) The relationship between TPX2 expression and the pathological stages of cancers. *p< 0.05, **p< 0.01, ***p< 0.001.

Next, we assessed the differential TPX2 expression based on clinical parameters. There was a higher expression of TPX2 in young patients in 9 types of tumors, while there were inconsistent results in ACC, LGG, PRAD, and UCEC (Figure 8A). TPX2 levels were lower in females in HNSC, KIRC, LUAD, LUSC, and SKCM than those in males, while females expressed higher levels in KIRP and SARC (Figure 8B). Ten types of tumors showed significant associations between TPX2 expression and tumor stage (Figure 8C).

### Correlation of TPX2 expression with tumor immune microenvironment

**Figure 9:**
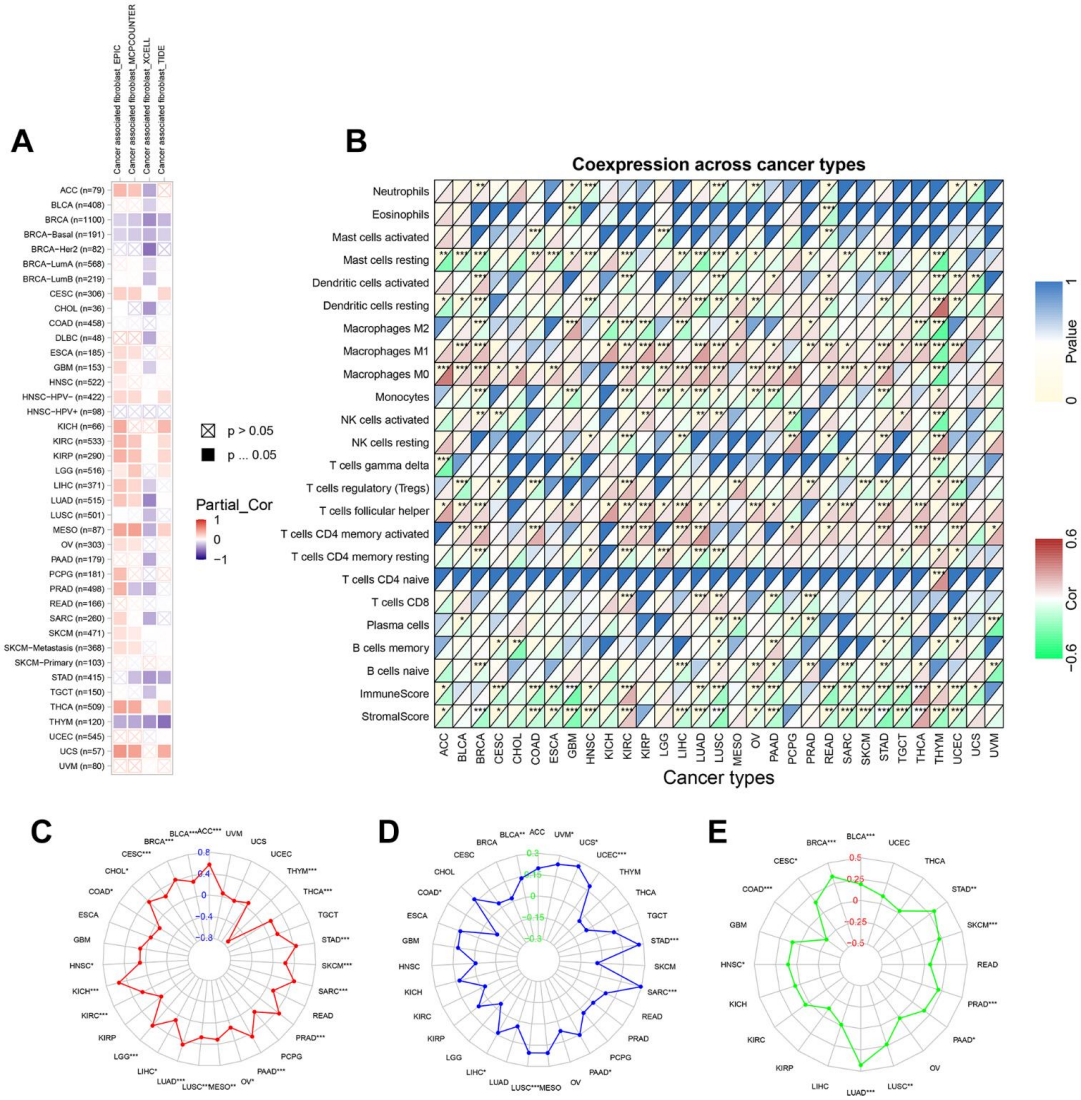
**The relationship between TPX2 expression and tumor microenvironment, TMB, MSI and neoantigens in various cancers from TCGA database.** (**A**) The relationship between TPX2 expression and CAF infiltration using the EPIC, MCPCOUNTEER, XCELL and TIDE algorithms. (**B**) The relationship between TPX2 expression and immune cell infiltration, immune score, and stomal score. (**C**) Radar map of association between TPX2 expression and TMB. (**D**) Radar map of association between TPX2 expression and MSI. (**E**) Radar map of association between TPX2 expression and neoantigens. *p<0.05, **p<0.01, ***p<0.001.

We explored the association of TPX2 expression and cancer-associated fibroblasts (CAF) infiltration using different algorithms. Negative correlations were found between TPX2 expression and CAF infiltration in BRCA and THYM at four different algorithms (Figure 9A). We then investigated possible associations of TPX2 expression with 22 immune cell subtypes infiltration using the Cell-type Identification by Estimating Relative Subsets of RNA Transcripts (CIBERSORT) algorithm. TPX2 expression was related to several different macrophage subpopulations. TPX2 expression and macrophage M1 infiltration were positively correlated in 17 cancer types, but negatively in THYM. Similarly, TPX2 expression and M0 macrophage levels were positively correlated in 19 cancer types, while a negative correlation in THYM. Additionally, TPX2 expression was negatively related to macrophage M2 levels in 7 cancer types, but positively in GBM, MESO, and PRAD. Further, TPX2 expression correlated with other immune cell levels in different cancer types. Next, using ESTIMATE, a negative association was observed between TPX2 expression and immune score and stromal score in 16 cancer types, but a positive association in KIRC and THCA (Figure 9B).

### Correlation analysis on TPX2 expression with TMB, MSI and neoantigens

We investigated the correlation of TPX2 with tumor mutational burden (TMB), microsatellite instability (MSI) and neoantigens. TPX2 had a positive correlation with TMB in 20 cancer types, while negative correlations were observed in COAD and THYM (Figure 9C). MSI had a positive association with TPX2 in 9 cancer types, but negatively associated with COAD (Figure 9D). TPX2 expression was positively related to neoantigens in 10 cancer types, but negatively in COAD (Figure 9E).

### Correlation of TPX2 expression with immune-related genes

**Figure 10:**
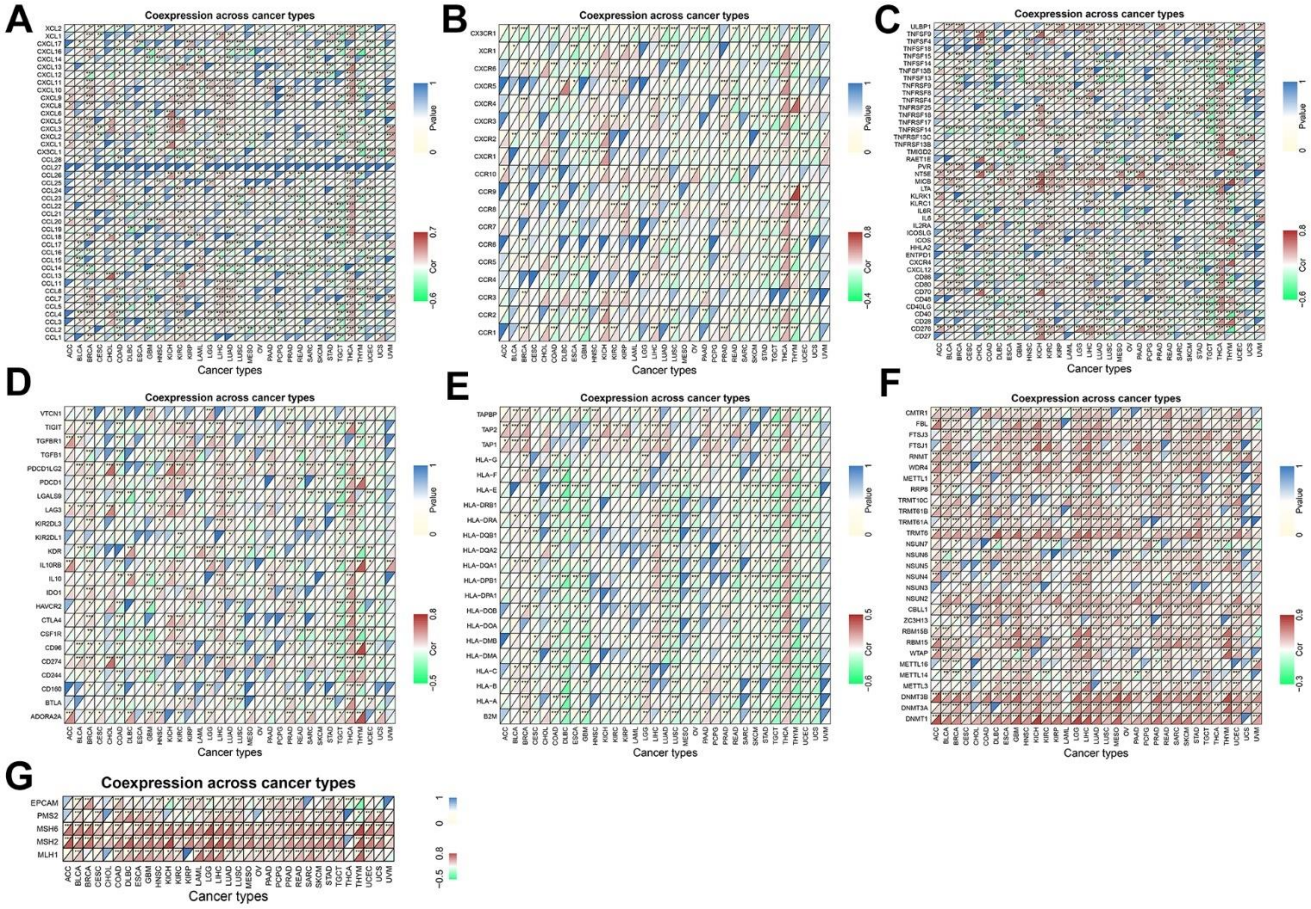
**Coexpression of TPX2 with immune-associated genes in 31 cancer types from the TCGA database.** (**A**) Heatmap of the association between TPX2 expression and chemokines. (**B**) Heatmap of the association between TPX2 expression and chemokine receptors. (**C**) Heatmap of the association between TPX2 expression and immune activation genes. (**D**) Heatmap of the association between TPX2 expression and immunosuppressive genes. (**E**) Heatmap of the association between TPX2 expression and MHC genes. (**F**) Heatmap of the association between TPX2 expression and DNA methyltransferases. (**G**) Heatmap of the association between TPX2 expression and MMRs genes.

Using gene co-expression analysis, further evaluation of the correlation between TPX2 and immune-related genes was conducted. Chemokines are signaling proteins essential for directing the migration of immune cells, thereby playing a pivotal role in immune responses. Additionally, they can modulate tumor growth and metastasis. Their receptors, present on a variety of cells, are instrumental in facilitating cellular migration and are critically implicated in both tumor progression and immune evasion [15]. We found that TPX2 expression correlated significantly with chemokine and chemokine receptor in almost all cancer types (Figure 10A, 10B). Positive relationship of immune activation genes and immunosuppressive genes with TPX2 was also found in multiple tumors (Figure 10C, 10D). MHC genes are instrumental in cancer immunity, displaying tumor-specific antigens to T cells and enhancing the immune system’s discernment of malignant cells. However, tumors can evade this system by downregulating MHC expression, rendering them less visible to immune surveillance. Levels of MHC expression can influence a tumor’s response to immunotherapies [16]. In our study, there was a negative correlation between TPX2 and MHC genes in most cancers (Figure 10E). DNA methylation, an epigenetic modification, plays a pivotal role in cancer progression. Methylation patterns of specific genes influence the tumor microenvironment, offer potential therapeutic targets, and serve as diagnostic or prognostic biomarkers [17]. Mismatch repair proteins (MMRs) are crucial for preserving DNA integrity during replication. Tumors exhibiting mismatch repair deficiency (dMMR) often possesses an elevated mutation burden. Notably, tumors with dMMR status frequently respond favorably to immunotherapies [18]. We found that DNA methyltransferases and MMRs genes were positively correlated with TPX2 in almost all cancer types (Figure 10F, 10G).

### Drug sensitivity analysis

**Figure 11:**
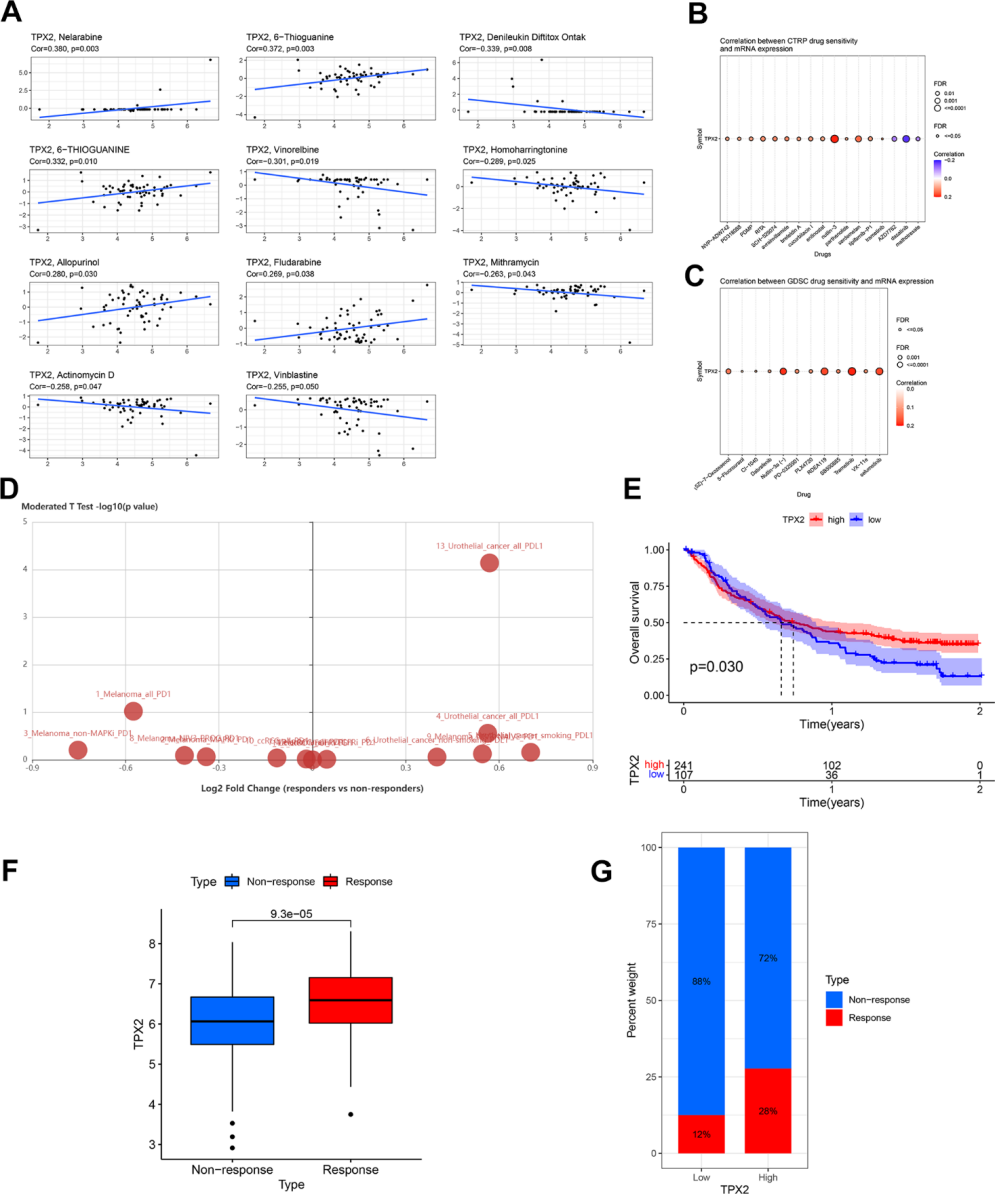
**Drug sensitivity analysis and validation of the immunotherapeutic predictive value of TPX2.** (**A**) Drug sensitivity analysis of TPX2 using the CellMiner database. (**B**) Correlation between CTRP drug sensitivity and TPX2 expression. (**C**) Correlation between GDSC drug sensitivity and TPX2 expression. Red dots indicate positive association. Blue dots indicate negative association. (**D**) Difference in expression of TPX2 between responders and non-responders undergoing anti-PD1/PD-L1 therapy using the TISIDB database. (**E**) Kaplan-Meier OS curves for TPX2 in IMvigor 210. (**F**) TPX2 expression was higher in responders than that in non-responders in IMvigor 210. (**G**) Treatment response rates with anti-PD-L1 therapy in patients with high and low expressions of TPX2 in IMvigor 210. p-value < 0.05 was considered statistically significant.

According to the CellMiner database, TPX2 mRNA expression was positively related to IC50 values of five drugs, but negatively related to six (Figure 11A). Patients with TPX2 elevated expression were likely to be sensitive to many anticancer drugs based on the Cancer Therapeutics Response Portal (CTRP) and Genomics of Drug Sensitivity in Cancer (GDSC) database (Figure 11B, 11C). In urothelial cancer, TPX2 expression was significantly different between responders and non-responders undergoing atezolizumab (anti-PD-L1) using the Tumor immune system interaction database (TISIDB) database (Figure 11D). Our findings can be used as a guide for selecting clinical therapeutic drugs.

### The prediction value of TPX2 to immunotherapy response

The above analysis indicated that TPX2 was related to tumor immune regulation. Then, we explored the prediction value of TPX2 to immunotherapy response according to IMvigor 210 cohort. Higher TPX2 expression was significantly related to longer OS (Figure 11E). Additionally, increased TPX2 levels were observed in responders (Figure 11F). Patients with high TPX2 levels displayed a better response to treatment with anti-PD-L1 (Figure 11G).

### Single-cell functional analysis of TPX2

**Figure 12:**
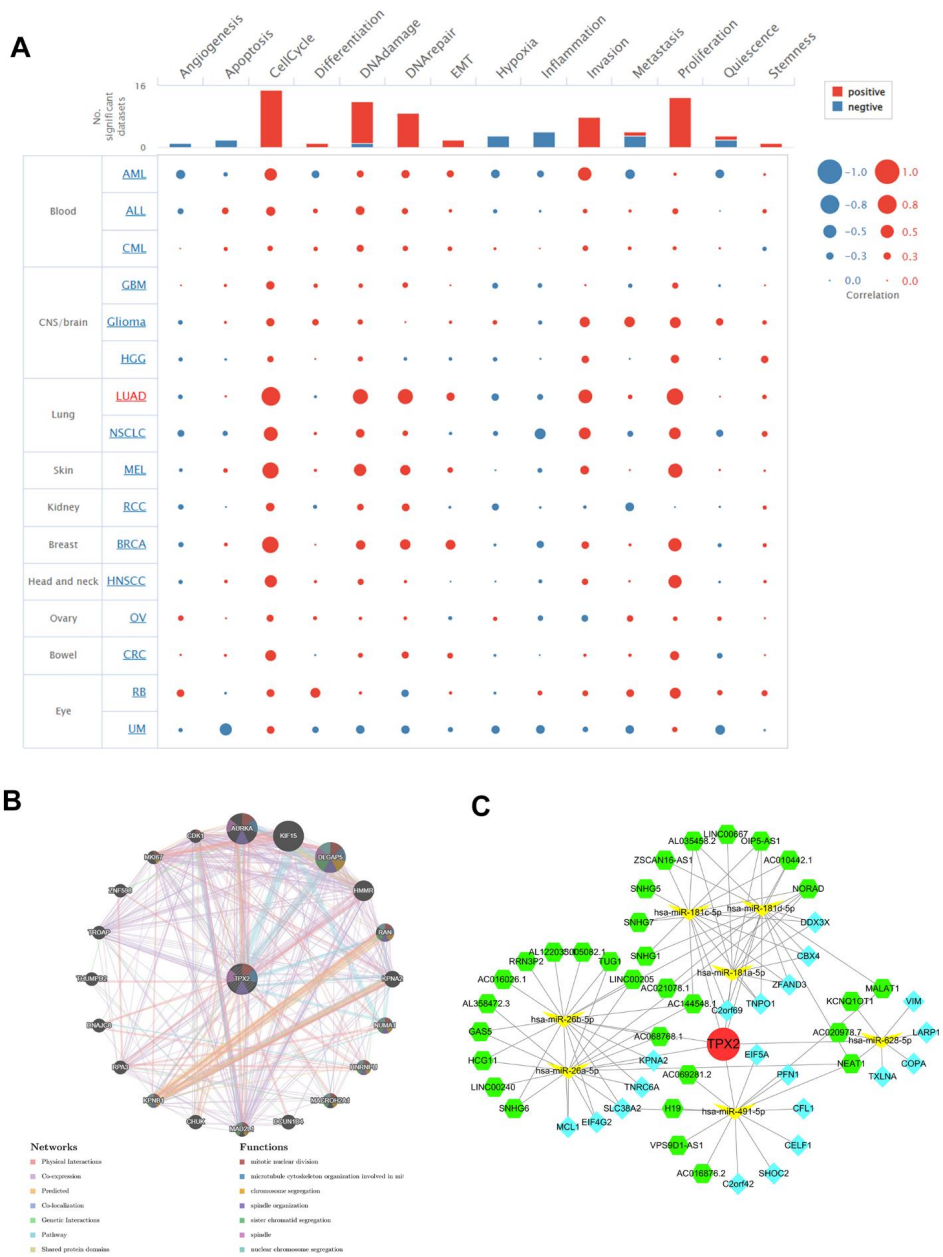
(**A**) Single-cell functional analysis of TPX2 from the CancerSEA database. (**B**) A gene-gene interaction network analysis of TPX2 from GeneMANIA database. (**C**) CeRNA networks of TPX2. Red circle represents the hub gene. Yellow vs represents the miRNAs. Green hexagons represent the lncRNAs. Blue quadrangles represent the circRNAs.

We used CancerSEA to investigate the correlation of TPX2 with 14 cancer functional states at the single-cell resolution. Multiple tumors, especially LUAD, showed positive correlations between TPX2 and cell cycle, DNA damage, DNA repair, invasion and proliferation (Figure 12A).

### Interacting genes of TPX2 and ceRNA network

TPX2 gene-gene interaction network was constructed using the GeneMANIA data. Twenty genes were closely related to TPX2, with AURAK, KIF15, and DLGAP5 showing the most significant correlations. In addition, according to the functional analysis, TPX2 and its similar genes were strongly related to the mitotic nuclear division, microtubule cytoskeleton organization and chromosome segregation (Figure 12B). The ceRNA network was constructed through the interaction of mRNAs, miRNAs, and their corresponding ncRNAs. A total of 7 target miRNAs of TPX2 were available in StarBase for circRNA and lncRNA prediction. In the ceRNA network, 31 lncRNAs, 20 cicRNAs and 7 miRNAs were included (Figure 12C).

### GSEA analysis

Gene set enrichment analysis (GSEA) was applied to explore the enrichment of Gene Ontology (GO) functional annotations and Kyoto Encyclopedia of Genes and Genomes (KEGG) pathway annotations in groups with high and low expression of TPX2 (Supplementary Figures 2, 3). In KEGG terms, TPX2 regulated “cell cycle” and “DNA replication” pathways in some tumors. In GO terms, TPX2 was mainly associated with the regulation of “leukocyte migration”, “antigen binding”, “complement activation” and “chromosome segregation”. Based on these findings, it is possible that TPX2 is involved in immune function and cell proliferation via certain signaling pathways.

### *In vitro* validation of TPX2 in lung cancer

**Figure 13:**
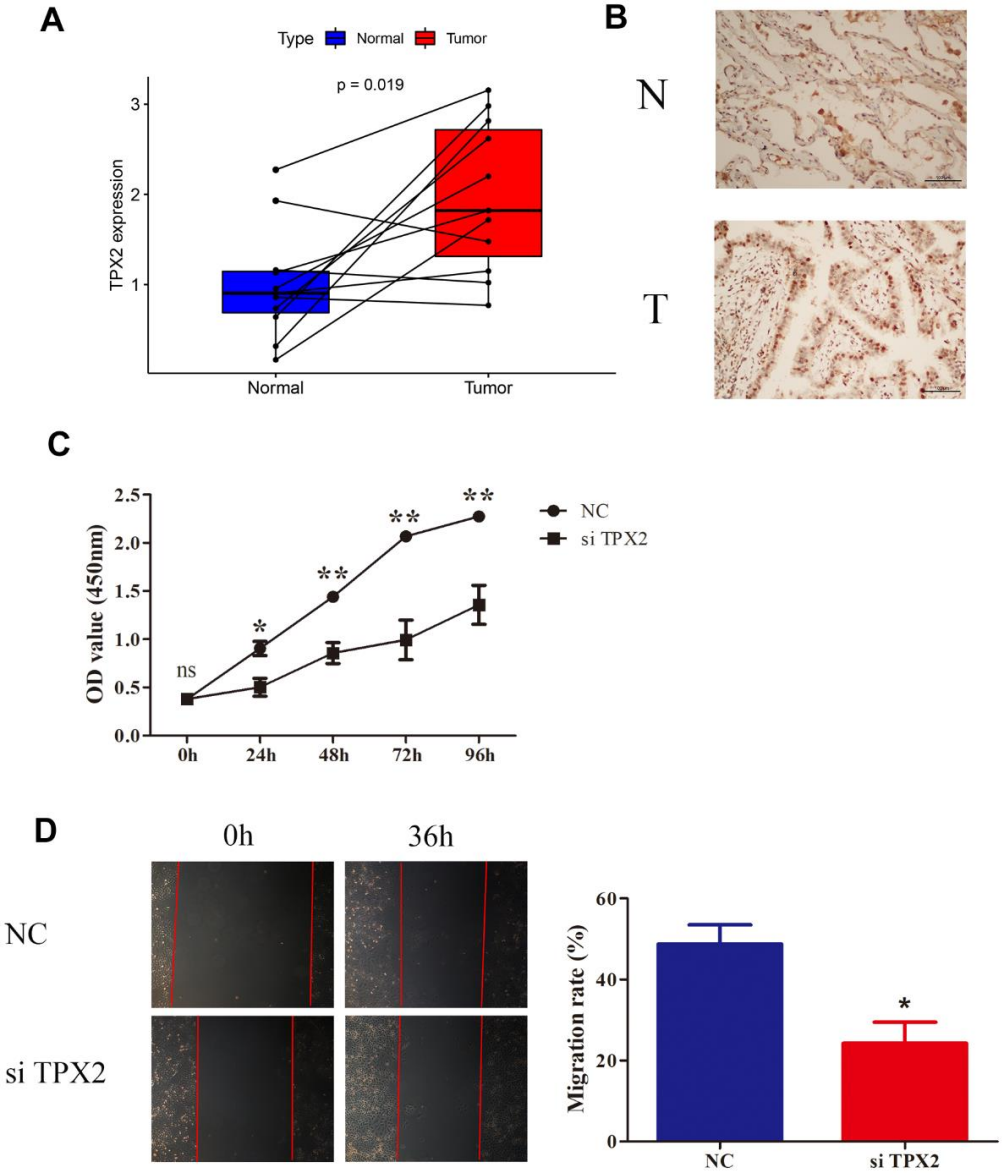
**Knockdown of TPX2 inhibited proliferation and migration of LUAD.** (**A**) TPX2 mRNA expression levels were higher in 11 LUAD tissues than that in matched normal tissue samples. Y-axis data presents relative expression (normalized to GAPDH; calculated using the 2^−ΔΔCt^ method). (**B**) Immunohistochemical analysis of TPX2 in LUAD tissues. Representative images are shown. (**C**) CCK8 assay suggested that knockdown of TPX2 inhibited the proliferation of A549 cells. (**D**) The wound healing assay suggested that knockdown of TPX2 reduced cell migration of A549 cells. *p<0.05, **p<0.01.

To validate TPX2 expression, LUAD tissues and normal tissues were collected in 11 pairs to detect TPX2 mRNA expression with qRT-PCR. As compared to normal tissues, LUAD tissues had significantly higher TPX2 expression (Figure 13A). According to immunohistochemistry, TPX2 was relatively overexpressed in LUAD, consistent with HPA database results (Figure 13B). To gain a deep understanding of the function of TPX2, lung cancer cell lines were chosen for study. siRNA targeting TPX2 was transfected into A549 cells to knock down endogenous TPX2. The CCK8 assay indicated that TPX2 knockdown reduced cell proliferation. The wound healing assay showed that TPX2 downregulation markedly reduced cell migration.

## DISCUSSION

The microtubule-linked protein TPX2 is closely associated with the cell cycle and plays a role in spindle assembly in human cells [1]. Moreover, TPX2 overexpression correlates with tumor proliferation, metastasis and poor outcomes in solid tumors [19–21]. For instance, TPX2 was reported to be a negative predictor for both DFS and OS in patients with HCC in which TPX2 stabilization via CDK5 could promote tumorigenesis [22]. Conversely, TPX2 downregulation could impede colon cancer and glioma cell proliferation and migration through PI3K/AKT/mTOR pathways [23, 24]. In breast cancer, TPX2 silencing could suppress proliferation and promote apoptosis through activating the p53 signaling pathway [25]. These previous studies have predominantly focused on TPX2 in individual cancer types. Emerging investigations have delved into pan-cancer studies, aiming to reveal common features and heterogeneities across different cancers, and thus aiding in the identification of novel therapeutic targets [26]. Our work delivers a holistic, pan-cancer view, bringing forth insights into TPX2’s role across an array of cancers, potentially highlighting commonalities and disparities among different cancer types. Beyond conventional expression profiling, we delved into multiple advanced analyses. Our study did not rely solely on bioinformatics. We conducted experimental validations and explored the biological functions of TPX2 in a lung cancer cell line, providing tangible evidence for our bioinformatics predictions.

The majority of cancers in our study showed up-regulation of TPX2 at mRNA and protein levels. Single-cell analysis revealed that TPX2 expression was up-regulated within malignant glioma cells. In addition, amplification emerged as the predominant genetic alteration of TPX2 in tumor cases. A negative correlation was observed between TPX2 methylation and TPX2 expression across multiple human cancers. In particular, hypomethylation occurred in BLCA, BRCA, HNSC, and LUAD. These findings suggested that genetic alteration and promoter methylation might contribute to the dysregulated expression of TPX2 in certain cancers. Utilizing the constructed ceRNA network, we further elucidated the potential upstream and downstream mechanisms for TPX2 expression. In certain cancers, TPX2 expression was also remarkably related to clinical stage, age, and gender. Particularly, some cancers showed different expression levels of TPX2 between stage I and IV underscoring pivotal roles of TPX2 in the development of cancers.

The ROC curve analysis indicated that TPX2 appeared to be an effective diagnostic marker. According to Cox regression and Kaplan-Meier analyses, upregulation of TPX2 correlated with an adverse prognosis in most cancers. The results were consistent with previous studies that proposed TPX2 as a negative prognostic biomarker [4–7]. Conversely, TPX2 had the opposite prognostic role in THYM, potentially due to its elevated expression levels in normal thymuses. Mutation analysis indicated that TPX2 mutation correlated with poor OS in UCEC, and worse PFS and DFS in LUSC. Our study demonstrated that TPX2 mRNA level was significantly upregulated in many types of cancers including LUAD. Additionally, TPX2 protein expression was also higher in some types of cancers including LUAD from the HPA cohort. Moreover, the results of prognosis analyses showed consistent results in LUAD. Kaplan-Meier analyses and Cox regression analyses demonstrated that higher TPX2 expression was associated with worse OS, DSS and PFI in LUAD. Due to consistent mRNA and protein expression of TPX2, as well as consistent clinical outcomes, we used LUAD as a bioinformatics validation example. To validate the expression of TPX2, qRT-PCR and immunohistochemistry were conducted in LUAD samples. LUAD exhibited a higher expression level of TPX2 at the mRNA and protein tiers than normal tissues, as corroborated by bioinformatics. Utilizing GeneMANIA analysis, GSEA analysis and single-cell functional analysis, TPX2 was primarily involved in proliferation, invasion, cell cycle, and DNA replication. Preliminary functional studies were performed on lung cancer cells to investigate the role of TPX2. The knockdown of TPX2 effectively reduced lung cancer cells proliferation, and invasion. The findings were consonance with bioinformatics, establishing the validity of our research.

Immune checkpoint inhibitors (ICIs) therapies have revolutionized the current therapeutic modalities of cancers. Nonetheless, there are still many patients who fail to respond [13, 14]. Reliable biomarkers are urgently needed to select patients who could benefit from immunotherapies. TMB is often quantified as the total number of mutations per coding area of a tumor genome. TMB-high tumors produce high levels of neoantigens, making them more immunogenic, and triggering a T cell response. Increasing evidence shows that high TMB is correlated with the effectiveness of ICI [27, 28]. The FDA has approved TMB as a genomic biomarker in some solid tumors [29]. Microsatellites, which are short tandem DNA repeats in the genome, exhibit variations in sequence lengths known as MSI due to insertions or deletions when compared to normal tissue. MSI is also burgeoning as a biomarker with predictive value in ICI therapies. It is increasingly evident that MSI-H was the consequence of mismatch-repair deficiencies (dMMR) and able to predict tumor development [30]. MSI-H/dMMR exhibited high mutation load and could act as an independent predictor of immunotherapy. MSI-H/dMMR was related to better response to ICI in most solid tumors [18, 31]. Neoantigens are a subset of antigens derived from protein-coding mutations unique to tumors. Immunotherapy efficacy could also be predicted by the neoantigens in the tumor microenvironment [32]. A high neoantigen load in tumors has been correlated with better responses to checkpoint inhibitors in some studies. The present investigation revealed that TPX2 was linked to TMB, MSI and neoantigens. These results suggested that TPX2 might modulate the TMB, MSI and neoantigens of most cancers, thereby influencing immunotherapy response.

Tumor infiltrating lymphocytes (TILs) within the tumor microenvironment have been related to prognosis in certain tumors. Recent studies posit that tumor-infiltrating lymphocytes possess the capacity to predict the efficacy of ICIs [33, 34]. Concurrently, emerging studies elucidate the predictive role of B cells for ICIs [35, 36]. In addition, tumor-associated macrophages (TAMs) contribute greatly to the efficacy of ICIs [37]. Nevertheless, the role of TPX2 in tumor immune landscape remains inadequately explored. Ahmed M Aref et al. showed that targeting TPX2 with specific peptides was able to improve the efficacy of cytotoxic T lymphocytes (CTLs) in T cell-mediated HCC immunotherapy [38]. Our findings revealed that multiple immune cell types were related to TPX2 expression. Within the IMvigor 210 cohort, 348 patients with metastatic urothelial bladder cancer were documented, among whom 78.2% exhibited resistance to cisplatin-based chemotherapy, with all undergoing atezolizumab treatment [39]. Our analysis based on IMvigor 210 indicated that patients with high TPX2 levels showed improved outcomes with anti-PD-L1 therapy. Furthermore, significant associations emerged between TPX2 expression and immune-related genes, particularly in BLCA, where TPX2 and CD274 (PD-L1) showed a positive correlation. Elevated TPX2 expression was positively associated with TMB and MSI in BLCA, potentially elucidating the enhanced immunotherapy response in patients with high TPX2 expression. Up-regulated TPX2 was significantly associated with worse clinical outcomes in most tumors from TCGA. The results from TCGA cohort and IMvigor 210 were not contradictory. On the contrary, it can be inferred that the patients who did not benefit from classic anticancer therapies might be responsible for the immunotherapy. These findings intimate a robust association between TPX2 and immune infiltration within tumors, influencing prognosis and providing a new immunotherapy biomarker.

Numerous clinical trials have shown that patients with elevated levels of PD-L1 expression in their tumors often exhibit better response rates to specific therapies than patients with minimal or absent PD-L1 expression [40]. However, it is not a flawless biomarker. Notably, some patients with PD-L1 negative tumor tissues have still shown responsiveness to PD-1/PD-L1 inhibitor treatments [41]. This can be attributed to the inducible nature of PD-L1 expression and its epigenetic modulation, as well as the spatial and temporal variability of PD-L1 expression within tumor tissues. The landscape of immune checkpoints in tumors is complex and dynamic. Tumors employ multiple immune evasion strategies, and they can exploit various checkpoints simultaneously or sequentially. CTLA-4 blockers, have shown efficacy even in cases where PD-L1 expression might be low [42]. Some tumors with low PD-L1 expression might indeed upregulate other immune checkpoints, like CTLA-4, LAG-3, or TIM-3 [43, 44]. The reasoning here is that these tumors might have adopted alternative immune evasion strategies that don’t heavily rely on the PD-L1/PD-1 axis. A tumor might also express multiple immune checkpoints concurrently. The expression of CTLA-4 on TILs was found to be enriched in PD-1+ T cells in a range of solid tumors [45]. Combining therapies targeting both PD-1/PD-L1 and CTLA-4 has shown synergistic effects in certain cancers, leading to enhanced anti-tumor responses compared to monotherapy [46].

## CONCLUSIONS

As shown in our study, we found significant upregulation of TPX2, alongside a negative association between expression levels of TPX2 and survival outcomes in a variety of tumors. Furthermore, infiltration of immune cells, immunotherapy response, gene alteration, DNA methylation, drug sensitivity and immune-related genes were correlated with aberrant TPX2 expression. However, the current study primarily hinges on bioinformatics without deep molecular mechanistic studies at cellular or animal levels. Moving forward, experimental validations of TPX2 in each tumor remain imperative. In summary, our study elucidates the potential of TPX2 as a diagnostic marker for cancer detection and classification, while also underscoring its utility as a prognostic indicator. Our findings underscore the capacity of TPX2 to guide the selection of patients likely to benefit from immunotherapies. Efforts could be directed toward developing small molecules, antibodies, or RNA-based therapeutics that target TPX2, and concurrently incorporating its use as a diagnostic and prognostic biomarker into clinical practice. By stratifying patients based on TPX2 expression levels, clinicians may facilitate more personalized and targeted therapeutic interventions. Our analysis establishes a solid foundation for exploring TPX2 as a promising target in cancer therapy.

## MATERIALS AND METHODS

### Data acquisition

We obtained RNA sequencing datasets for 31 solid cancer types, including raw counts and Fragments per Kilobase of Transcript per Million (FPKM)-normalized data, as well as relevant clinical information from TCGA data portal (https://portal.gdc.cancer.gov/) on March 20 2022. Table 1 presented the primary information about 31 types of solid tumors. From projects of the HPA (https://www.proteinatlas.org/), we acquired the distribution of TPX2 protein at the subcellular level and immunohistochemistry images of TPX2. We obtained transcriptomic and clinical data from IMvigor 210 for BLCA undergoing anti-PD-L1 therapy (atezolizumab) [39]. R version 3.6.3 was used for data analysis.

**Table 1:** Pan-cancer data acquired from TCGA.

**Cancer type**	**Full name**	**Tumor samples**	**Normal samples**
ACC	Adrenocortical carcinoma	79	0
BLCA	Bladder urothelial carcinoma	408	19
BRCA	Breast invasive carcinoma	1091	113
CESC	Cervical squamous cell carcinoma and endocervical adenocarcinoma	304	3
CHOL	Cholangiocarcinoma	36	9
COAD	Colon adenocarcinoma	456	41
ESCA	Esophageal carcinoma	161	11
GBM	Glioblastoma multiforme	161	5
HNSC	Head and neck squamous cell carcinoma	500	44
KICH	Kidney chromophobe	65	24
KIRC	Kidney renal clear cell carcinoma	530	72
KIRP	Kidney renal papillary cell carcinoma	288	32
LGG	Brain lower grade glioma	511	0
LIHC	Liver hepatocellular carcinoma	371	50
LUAD	Lung adenocarcinoma	513	59
LUSC	Lung squamous cell carcinoma	501	49
MESO	Mesothelioma	86	0
OV	Ovarian serous cystadenocarcinoma	376	0
PAAD	Pancreatic adenocarcinoma	177	4
PCPG	Pheochromocytoma and paraganglioma	179	3
PRAD	Prostate adenocarcinoma	495	52
READ	Rectum adenocarcinoma	166	10
SARC	Sarcoma	259	2
SKCM	Skin cutaneous melanoma	468	1
STAD	Stomach adenocarcinoma	375	32
TGCT	Testicular germ cell tumors	150	0
THCA	Thyroid carcinoma	502	58
THYM	Thymoma	119	2
UCEC	Uterine corpus endometrial carcinoma	543	35
UCS	Uterine carcinosarcoma	56	0
UVM	Uveal melanoma	80	0

### Expression and diagnosis analysis of TPX2

TPX2 mRNA differential expression was explored using Wilcoxon test. We applied ROC curve to detect the diagnostic value of TPX2. The AUC value of >0.7 was considered to be a good diagnostic value. The chi-square test or Fisher’s exact test was used to investigate the relationship of TPX2 expression with other clinical parameters.

### Single-cell analysis of TPX2

The Tumor Immune Single-cell Hub (TISCH) web tool was used for single-cell analysis [47]. Heatmaps, scatter diagrams, and violin plots were applied to quantify and visualize TPX2 expression in each cell type. We explored the functional states of TPX2 using single-cell sequence data obtained from the CancerSEA website (biocc.hrbmu.edu.cn/CancerSEA/) in the “correlation plot” module [48].

### DNA methylation and mutation status analysis

We applied GSCA (http://bioinfo.life.hust.edu.cn/GSCA/#/) to identify the correlations between TPX2 DNA methylation levels and TPX2 expression, and the difference of TPX2 methylation in cancer tissues and paired normal tissues. The correlation between TPX2 expression and CNV was also explored using GSCA [49]. The public database cBioPortal (https://www.cbioportal.org/) was applied to analyze TPX2 alterations in the TCGA pan-cancer samples [50].

### Protein phosphorylation analysis

The level of phospho-TPX2 in the normal and primary tumor tissues was explored based on CPTAC analysis of the UALCAN portal (RRID: SCR_015827). Results were shown using the normalized Z value. We identified prominent phosphorylation sites on TPX2 integral domains using NCBI (https://www.ncbi.nlm.nih.gov/) and IBS (http://ibs.biocuckoo.org/).

### Survival analysis

Survival information was obtained from the TCGA. Using Kaplan-Meier and Cox proportional hazards analysis, the association of TPX2 expression level and survival outcomes was explored. Based on the median level of TPX2, each sample was classified either as high or low expression group. The forest plots and Kaplan–Meier were conducted by “survival”, “survminer” and “forestplot” R packages.

### Immune correlation analysis

We applied the EPIC, MCPCOUNTEER, XCELL and TIDE algorithms to assess the correlation between TPX2 expression and the infiltration of CAF [51]. Based on the CIBERSORT algorithm, we evaluated the relationship between TPX2 and 22 immune cell subtypes [52]. To investigate the abundance of immune and stromal components, stromal and immune cell scores are used, which are calculated based on ESTIMATE algorithm using R packages ‘estimate’ and ‘limma’ [53]. Recent studies indicate that TMB, MSI and neoantigens are biomarkers that can be used to monitor immune response [54]. We acquired somatic mutation data from 31 types of tumors in TCGA. Then, TMB scores were calculated by Perl language in each sample [27, 28]. MSI scores were obtained by MANTIS [30]. Using The Cancer Immunome Atlas (TCIA), we acquired neoantigen counts on each tumor sample cell separately [55]. Next, we analyzed the relationship of TMB, MSI and neoantigen with TPX2 based on Spearman correlation analysis. The association of TPX2 expression with immune-related genes was also analyzed. The results were displayed in heatmaps by applying ‘R Color Brewer’ and ‘Reshape 2’ packages.

### Drug sensitivity analysis

TPX2 mRNA expression in NCI-60 cell lines data and drug sensitivity data were acquired from CellMiner (http://discover.nci.nih.gov/cellminer) [56]. We also obtained drug sensitivity data from the CTRP and GDSC database. The correlation of TPX2 mRNA expression with drug sensitivity was evaluated by Pearson correlation analysis. We compared the TPX2 expression difference between responders and non-responders receiving PD1/PD-L1 inhibitor using the TISIDB database [57].

### GeneMANIA analysis and construction of ceRNA network

The GeneMANIA database (http://www.genemania.org) was used for the construction of the TPX2 interaction network. The network analysis performed using GeneMANIA was based on data specifically from *Homo sapiens* [58]. We predicted the potential miRNAs of TPX2 from PITA, RNA22, miRmap, DIANAmicroT, miRanda, PicTar, and TargetScan. Those miRNAs identified in three or more databases were considered target miRNAs. StarBase v2.0 was (https://starbase.sysu.edu.cn) used for the construction of miRNA–lncRNA interactions and miRNA–circRNA interactions. These results were confirmed using Ago CLIP-seq data [59]. The ceRNA networks were visualized using the Cytoscape.

### Gene set enrichment analysis

To investigate the functions of TPX2, GSEA was conducted based on ‘cluster-Profiler’ R package by GO and KEGG. We considered significant enrichment when gene sets fulfilled certain criteria (|NES|>1, NOM p<0.05, and FDR q<0.25).

### RNA isolation and quantitative real-time PCR analysis

LUAD tissues were obtained from First Affiliated Hospital of Nanjing Medical University. TRIzol reagent (Invitrogen, USA) was applied to extract total RNA according to the manufacturer’s instructions. With PrimeScript RT reagent Kit (Takara Bio), cDNA was synthesized. Then, qRT-PCR for TPX2 mRNA (forward: 5’ -TTCAAGGCTCGTCCAAACACCG -3’ and reverse: 5’ - GCTCTCTTCTCAGTAGCCAGCT-3’) was performed with an ABI StepOnePlus system (Applied Biosystems). GAPDH mRNA (forward: 5’ -GTCTCCTCTGACTTCAACAGCG -3’ and reverse: 5’ - ACCACCCTGTTGCTGTAGCCAA-3’) was used as the internal reference. 2^-ΔΔCt^ method was used to evaluate relative expression of TPX2 mRNA.

### Immunohistochemistry

Archival formalin fixed paraffin-embedded (FFPE) specimens of LUAD tissue samples and adjacent normal tissue samples were acquired from First Affiliated Hospital of Nanjing Medical University. FFPE tissue blocks were cut into four μm sections. Primary antibodies (TPX2, no., bs-4285R; dilution, 1:200; MA, USA) were incubated overnight at 4° C. A microscope (Olympus Corporation, Tokyo, Japan) was used to photograph immunostained sections.

### Cell culture

The human lung cancer cell lines A549 were acquired from the National Institute of Cells (Shanghai, China). All of the cells were cultured in RPMI-1640 medium containing 10% fetal calf serum (Gibco; USA) and maintained in a humidified atmosphere with 5% CO2 at 37° C.

### siRNA transfection

The siRNA targeting TPX2 were obtained from ShanghaGenePharma Co., Ltd., (Shanghai, China): Sense, 5’-CCA UUA ACC UGC CAG AGA AT-3’ and antisense, 5’-UUC UCU GGC AGG UUA AUG GT-3’. A negative control siRNA (si-NC) was also acquired: Sense, 5’-UUC UCC GAA CGU GUC ACG UTT-3’ and antisense, 5’-ACG UGA CAC GUU CGG AGA ATT-3’. TPX2 siRNA or si-NC was transfected into A549 cells using Lipofectamine 2000 (Thermo Fisher Scientific, USA) according to the manufacturer’s instructions.

### Cell viability assay

Lung cancer cell viability was examined using CCK8 assay kit according to the manufacturer’s instructions. Transfected A549 cells were plated into 96-well plates at a density of 2×10^3^ cells/well. The absorbance value of each well was read at 450 nm.

### Wound healing assay

We incubated the transfected A549 cells until they reached 100% confluency. With a 200 μl pipette tip, scraped cells in a straight line and rinsed with PBS three times. At 0 and 24h after incubation, the cell migration data were acquired using an inverted microscope.

### Statistical analysis

Statistical analysis of the experiments was performed using SPSS 20.0. Data are presented as the mean ± standard deviation (SD) from at least three separate experiments and analyzed using the Student’s t-test.

### Data availability

All data included in this study are available by contacting the corresponding authors.

## Supplementary Material

Supplementary Figures
