# Supplementary material for: Integrative analysis confirms TPX2 as a novel biomarker for clinical implication, tumor microenvironment, and immunotherapy response across human solid tumors
Source: Aging (Albany NY). 2024 Feb 2;16(3):2563–90. doi: 10.18632/aging.205498 (PMC10911359; doi:10.18632/aging.205498)
Supplement: Supplementary Figures [file aging-16-205498-s001.pdf]

SUPPLEMENTARY FIGURES

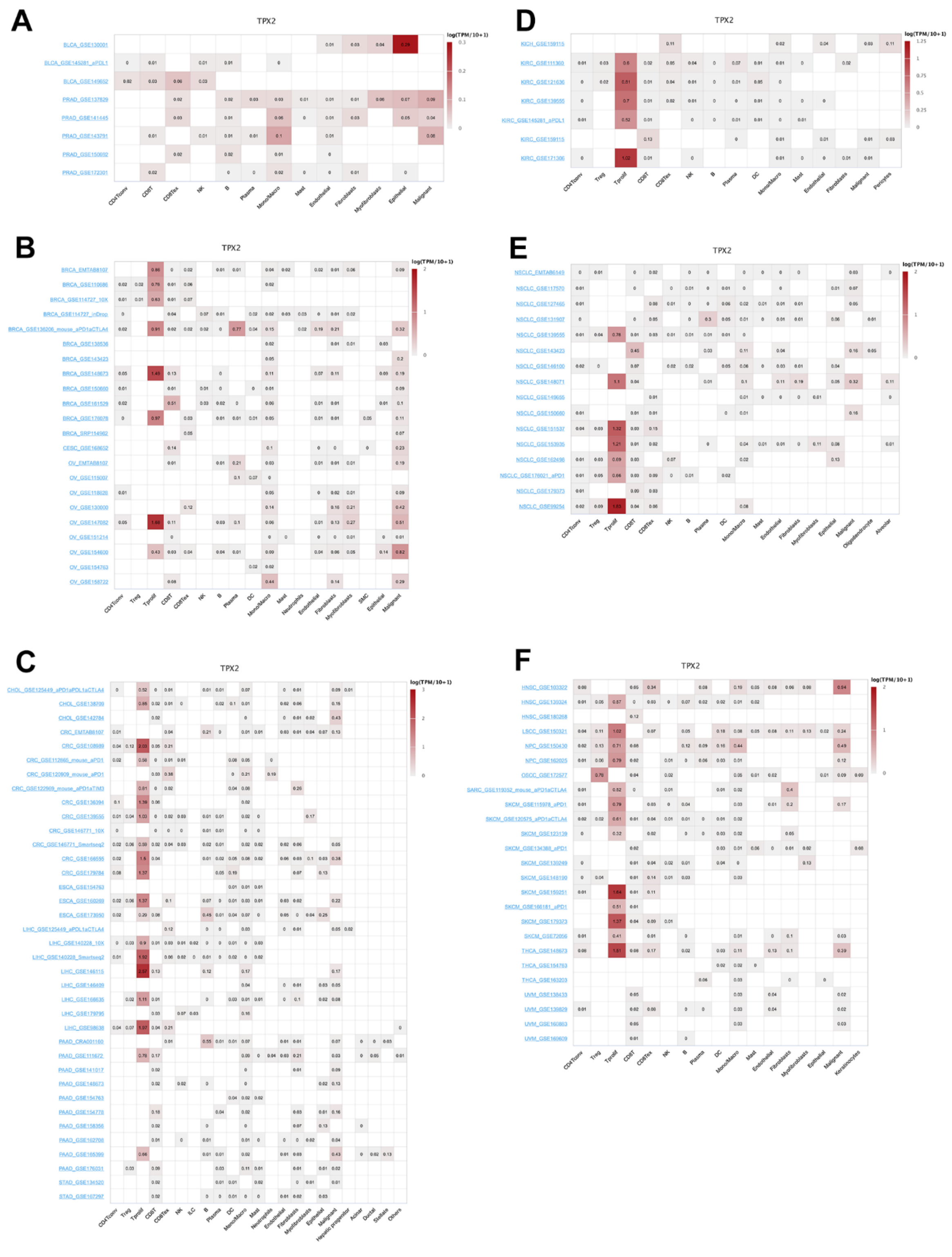

Supplementary Figure 1. Single-cell expression analysis of TPX2 in diverse cell types in genitourinary cancer (**A**), gynecologic tumors (**B**), digestive system cancer (**C**), renal cancer (**D**), NSCLC (**E**), and other types of cancer (**F**) in TISCH database.

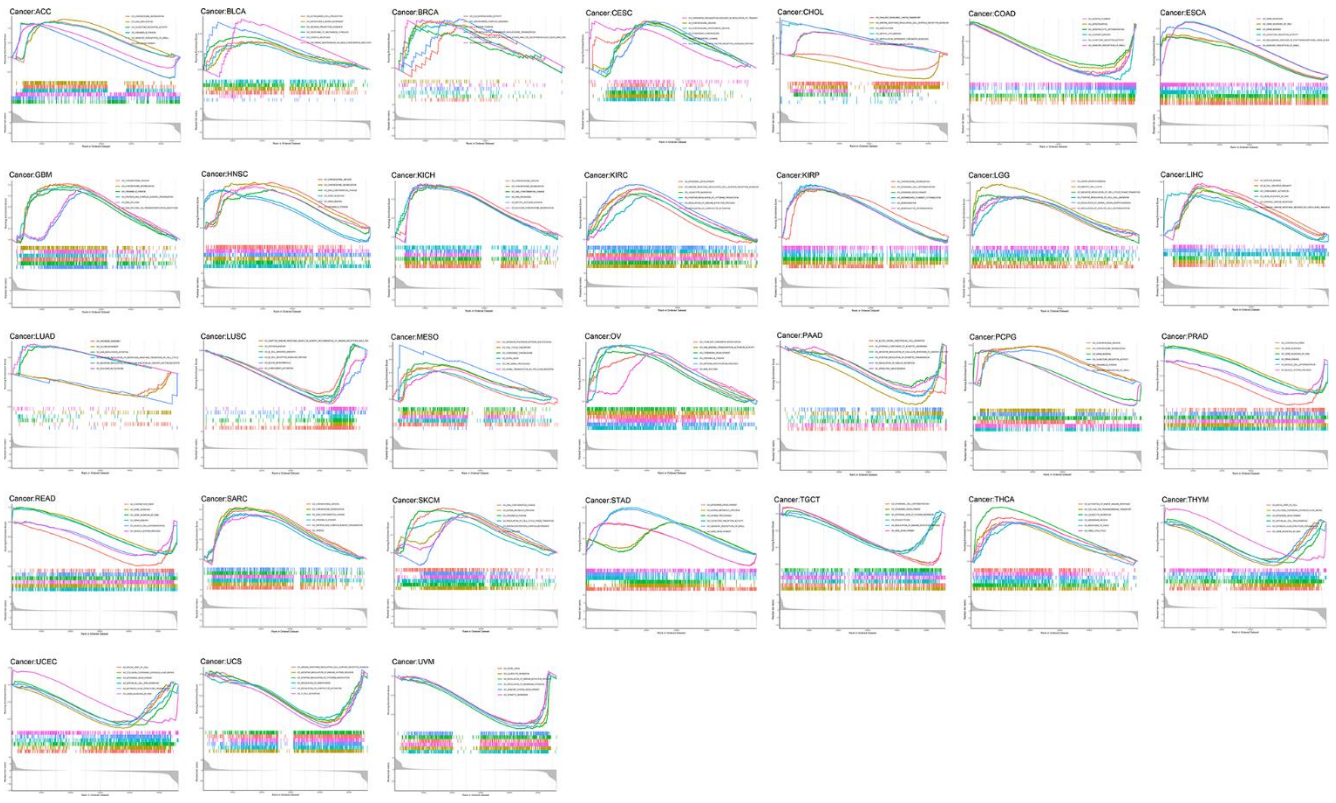

Supplementary Figure 2. Results of GSEA for TPX2 correlation with signaling pathways in GO collection.

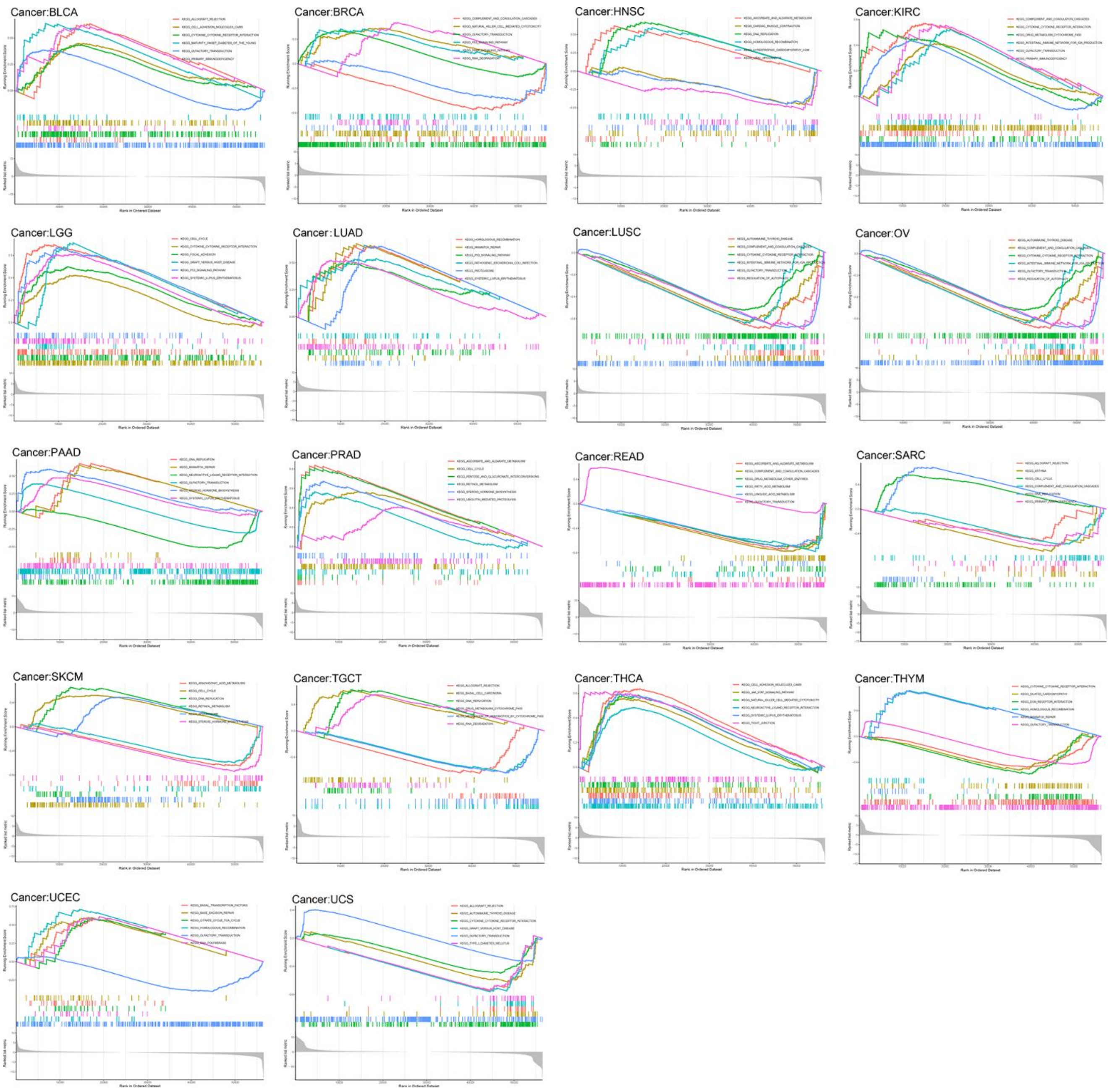

Supplementary Figure 3. Results of GSEA for TPX2 correlation with signaling pathways in KEGG collection.
